# Factors Influencing the Formation of Salicylic Acid by Bipolar Membranes Electrodialysis

**DOI:** 10.3390/membranes12020149

**Published:** 2022-01-26

**Authors:** Juan Taumaturgo Medina-Collana, Jimmy Aurelio Rosales-Huamani, Elmar Javier Franco-Gonzales, Jorge Alberto Montaño-Pisfil

**Affiliations:** 1 Faculty of Chemical Engineering, National University of Callao, Juan Pablo II 306 Avenue, Bellavista, Callao 07011, Peru; jtmedinac@unac.edu.pe (J.T.M.-C.); jamontanop@unac.edu.pe (J.A.M.-P.); 2 Multidisciplinary Sensing, Universal Accessibility and Machine Learning Group, Faculty of Geological, Mining and Metallurgical Engineering of the National University of Engineering, Lima 15333, Peru; efrancog@uni.edu.pe

**Keywords:** bipolar membrane, electrodialysis, taguchi method, salicylic acid

## Abstract

Salicylic acid is an intermediate product in the synthesis of dyes, medications and aspirin. An electrodialysis module has been constructed with commercial cationic, anionic and bipolar membranes for the conversion of sodium salicylate into salicylic acid. The effect of operating conditions such as applied electric potential, salt concentration, initial acid concentration and volumetric flow on bipolar membrane electrodialysis (BMED) yields were investigated using Taguchi analysis. The results obtained in 210 min of work show an average concentration of salicylic acid of 0.0185 M, an average electric current efficiency of 85.3%, and a specific energy consumption of 2.24 kWh/kg of salicylic acid. It was concluded that the proposed bipolar membrane electrodialysis process is an efficient alternative to produce salicylic acid (SAH) from sodium salicylate (SANa) in an environmentally friendly manner. Furthermore, the production of sodium hydroxide was obtained as a by-product of the process carried out.

## 1. Introduction

Salicylic acid or 2-hydroxybenzoic acid is widely distributed in the plant kingdom [1], and is most used for its analgesic, antipyretic and anti-inflammatory properties [2]. Salicylic acid (SAH) is an important raw material that has been used as an intermediate to synthesize drugs (such as aspirin) and cosmetics (used for the treatment of seborrheic dermatitis) [3].

**Figure 1:**
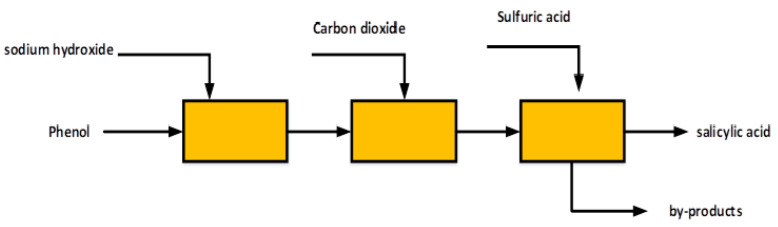
Production of salicylic acid by the Kolbe–Schmitt reaction.

Currently, the production of salicylic acid is based on Kolbe-Schmitt [4]. First, sodium phenolate is synthesized by the reaction between phenol and NaOH. Second, the intermediate is transformed into sodium salicylate (SANa) by a carboxylation reaction under conditions of high pressure, high temperature and CO${}_{2}$ atmosphere. Third, salicylic acid is obtained by acidification using a huge amount of H${}_{2}$SO${}_{4}$. However, this process will not only consume a considerable amount of H${}_{2}$SO${}_{4}$, but will generate numerous Na${}_{2}$SO${}_{4}$ waste solutions with a concentration greater than 0.1% *w*/*w* [5]. Figure 1 shows the block diagram of the process for obtaining salicylic acid.

**Figure 2:**
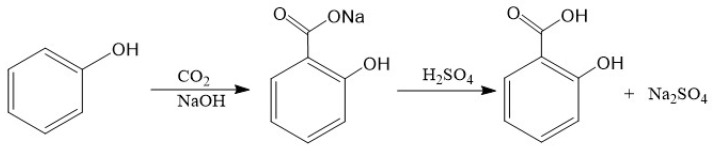
The Kolbe-Schmitt reaction.

The production of salicylic acid is based on the Kolbe-Schmitt reaction by the following reaction, shown in Figure 2.

As an advanced technical and environmentally friendly process, bipolar membrane electrodialysis (BMED) has been used as an alternative for the production of salicylic acid [5]. Electrodialysis technology has been applied to the treatment of high salt wastewater on an industrial scale since the 1950s [6]. Furthermore, the use of a bipolar membrane was introduced into the process in the 1970s, forming a new technology, electrodialysis with bipolar membranes (BMED), and expanding the application of this approach [7]. Among membrane technologies, electromembrane processes are promising technologies for the recovery of organic acids [8]. Electrodialysis (ED) is a separation process based on ion exchange membranes where positive ions are transported through a cation exchange membrane (CEM), and negative ions are transported through an anion exchange membrane (AEM) due to an applied electric field [9]. Electrodialysis (ED) allows the concentration and separation of cations/anions using cation exchange membranes (CEM) and anion exchange membranes (AEM). Furthermore, electrodialysis with bipolar membranes (BMED) is used to produce acids and bases from the corresponding salts [10,11].

Bipolar membrane electrodialysis technology (BMED) can be considered as a combination of a cation exchange membrane (CEM) and an anion exchange membrane (AEM); however, it has a different function than unipolar membranes [12]. BMED technology has been applied to the production of propionic acid [13], salicylic acid [14], gluconic acid [15] and other organic acids. The BMED dissociates water into protons and hydroxide, which move through the cation and anion layers of the bipolar membrane (FBM), respectively, and thus produce acidic and alkaline currents [16,17].

Independently, BMED has also been used effectively to produce or purify acids, as well as to adjust pH during fermentation or chemical synthesis in food and biochemical processing [18,19]. The BMED process has also been widely used for many years in the recovery of process water [20]. In [21], they used bipolar membranes for internal pH control in the electrodialysis of amino acids. Furthermore, [22] mentioned that the BMED process is a promising technology for the treatment of textile wastewater, as it produces acid and base from the salt content of the wastewater. In addition, it does not produce any waste stream that needs further treatment. Then, [23] observed that the maximum acid-base conductivity (90,000 µS/cm in acid, 60,000 µS/cm in base) was reached with the BMED system using only the salt in the concentrate. Relating concentrations correspond to approximately 1.44% acids(HCl) and 2% bases(NaOH). Independently, [24] suggested an economic value of BMED for desalination of high salinity textile wastewater.

When an electric field is applied to the BMED cell, the following cathodic and anodic reactions take place at the related electrodes, [25] as shown in Equations (1) and (2).

(1) $$\mathrm{cathodic}\;\mathrm{reaction}:\;\;\;\;\;\;2H_{2}O+2e^{-}\to 2{\mathrm{OH}}^{-}+H_{2}$$

(2) $$\mathrm{anodic}\;\mathrm{reaction}:\;\;\;\;\;\;\;2H_{2}O-2e^{-}\to 4H^{+}+O_{2}$$

The water molecules that diffuse in the bipolar membrane are divided into ions H${}^{+}$ and OH${}^{-}$ at the bipolar membrane interface both by the applied electric field and by the catalytic effect of the bipolar junction [26] as shown in Equation (3).

(3) $$\mathrm{bipolar}\;\mathrm{membrane}:\;\;\;\;\;H_{2}O\to {\mathrm{OH}}^{-}+H^{+}$$

The H${}^{+}$ ions pass through the cation exchange layer of the BPM towards the cathode side, while the OH${}^{-}$ ions pass through the anion exchange layer towards the anode side.

The salts in the feed stream ionize in aqueous medium as shown in Equation (4). The cation and anion exchange membranes change the direction of the ions to the appropriate compartments under the effect of the electric field.

(4) $$C_{7}H_{5}O_{3}\mathrm{Na}\to C_{7}H_{5}O_{3}^{-}+{\mathrm{Na}}^{+}$$

The C${}_{7}$H${}_{5}$O${}_{3}^{-}$(SA${}^{-}$) ions released migrate through the AEM and combine with the H${}^{+}$ ions produced by the dissociation of water from the bipolar membrane to form pure salicylic acid (acid compartment), as shown in Equation (5).

(5) $$C_{7}H_{5}O_{3}^{-}+H^{+}\to C_{7}H_{5}O_{3}H$$

Meanwhile, the released Na${}^{+}$ ions migrate through the CEM and combine with the OH${}^{-}$ ions produced by the dissociation of water to form pure sodium hydroxide (base compartment) [8], as shown in Equation (6).

(6) $${\mathrm{Na}}^{+}+{\mathrm{OH}}^{-}\to \mathrm{NaOH}$$

As an environmentally friendly and technical advanced process, bipolar membrane electrodialysis (BMED) has been used as an alternative for the production of salicylic acid [5].

The contribution of the work is the implementation, construction and evaluation of its own bipolar electrodialysis module. From there, the present study aimed to study the feasibility of the bipolar membrane electrodialysis process, and to evaluate the influence of operating factors during the bipolar electrodialysis process of the sodium salicylate salt in the formation of salicylic acid. Process parameters such as applied voltage, salt concentration, initial acid concentration, and flow affecting BMED have been evaluated. The result of this research shows the performance of the BMED process in the synthesis of salicylic acid and its influence on the operating variables of the equipment with respect to the acid concentration.

The present work is divided as follows: in Section 2 we mention the materials and methods to be used, in Section 3 we present the results and discussions carried out, and finally in Section 4 we discuss the conclusions obtained from this study.

## 2. Materials and Methods

### 2.1. Materials

The chemicals used in our research were analytically pure sodium salicylate salt, sodium hydroxide (NaOH), sodium sulfate, and phenolphthalein. Deionized water with a concentration of 1 ppm was used for the analysis. The glass materials used were burettes to carry out the titration operation.

### 2.2. Membranes

The experiment involved the use of three types of membranes, cation exchange (CEM), anion exchange (AEM), and bipolar membrane (FBM) were supplied by the company (Fumatech Bwt GmbH, Bietigheim Bissingen, Germany) for the study. For a good functioning of the membrane, it is first immersed in distilled water for a period of 24 h and then in an aqueous solution of NaCl 0.25 N for the other 24 h; the characteristics are shown in Table 1.

For FBM, the membrane area resistance was measured as Cl${}^{-}$ and Na${}^{+}$ in 0.5 M NaCl solution and 0.25 M Na${}_{2}$SO${}_{4}$ electrode rinse solution at 25 ${}^{\circ }$C, respectively [28].

### 2.3. Chemical Analysis

The changes in the concentration of salicylic acid and sodium hydroxide were determined by acid-base titration with a calibrated solution of 0.01 N NaOH and 0.01 N HCl using phenolphthalein as an indicator. The salicylic acid concentration was calculated by Equation (7) [14].

(7) $$C_{\mathrm{AS}}=\frac{C_{\mathrm{NaOH}}V_{\mathrm{NaOH}}}{V_{\mathrm{AS}}}$$

C${}_{\mathrm{NaOH}}$ (mol/L): concentration of the calibrated NaOH solution.

V${}_{\mathrm{NaOH}}$ (L): volume of the NaOH.

V${}_{\mathrm{AS}}$ (L): volume of acid used for titration.

C${}_{\mathrm{AS}}$: obtained concentration of salicylic acid.

### 2.4. Membrane Configuration

**Figure 3:**
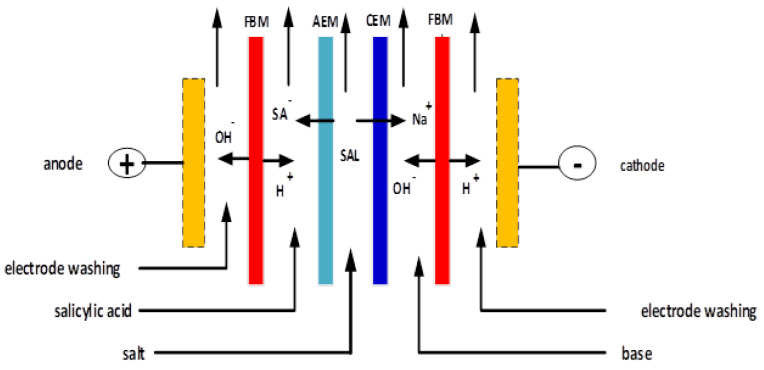
Schematic diagram of an BMED stack.

The BMED technology has been developed as a sustainable approach to split an aqueous saline stream into its corresponding acid and base without any use of chemicals [7]. BMED is configured with a series of ion exchange membranes, including anion exchange membranes (AEM), cation exchange membranes (CEM), and bipolar membranes (FBM) between a pair of electrodes [29]. This is shown in Figure 3.

The transport of water by osmosis in the BMED compartments is not within the scope of this work. However, ion exchange membranes in electrodialysis should possess high perm selectivity and conductivity, low resistance, and high mechanical, dimensional, and chemical stability [9,30,31].

### 2.5. Electrodialysis Cell with Bipolar Membrane

**Figure 4:**
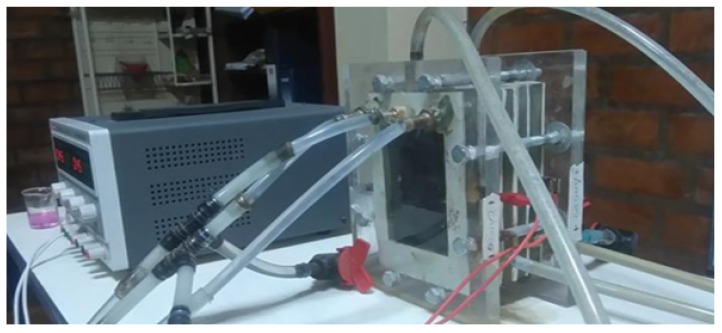
Filter press type.

**Figure 5:**
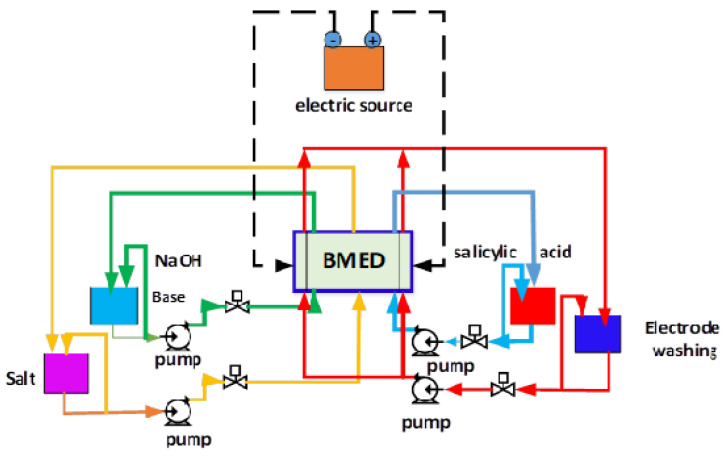
Electrodialysis cell with bipolar membrane.

The bipolar membrane electrodialysis cell is a filter press type as shown in Figure 4, with five compartments (acid, salt, base and washing of cathode electrodes and washing of anode electrodes). It was built with acrylic material on which four membranes are assembled with their respective mesh-shaped turbulence promoters, alternately fixed and secured with eight cross bars with nuts to prevent leakage, mixing or spilling of the liquid as shown in Figure 5.

Rubber gaskets were placed between each frame and membrane that allows a seal in order to maintain uniform flow distribution within the cells, with two acrylic plates at the ends of each, with three inlets and three outlets for the flow of solutions.

The external acrylic plates allow the system to be kept under pressure, providing stability and resistance to the set of cells. The the electrodes are inserted into two 1.5 cm thich acrylic frames, each with an inlet and an outlet through which the electrode wash solution enters.

In all compartments, solutions were circulated in a batch mode using pumps; changes in concentration were measured by sampling at time intervals.

A direct current generator supplied a constant current. The titanium electrode, both anode and cathode, has a working area of 100 cm${}^{2}$, and each membrane has an effective area of 90 cm${}^{2}$ (total effective membrane area of 360 cm${}^{2}$). The experiments were carried out at a room temperature of 28 ${}^{\circ }$C. As electrode washing solution, 2 L of 0.25 M sodium sulfate was used, the initial concentration of sodium hydroxide in each experiment being 0.01 N.

### 2.6. Calculation of Current Efficiency and Energy Consumption

The performance of BMED for salicylic acid synthesis was evaluated in terms of energy consumption (E), and current efficiency (η).

The current efficiency (η) is important for the characterization of the bipolar electrodialysis process. It was calculated by Equation (8) [32].

(8) $$\eta =\frac{z(C_{t}-C_{0})VF}{NIt}\times 100\%$$

$C_{t}$ and $C_{0}$ (mol/L): are the concentrations of salicylic acid at *t* and zero time respectively.

V(L): is the initial volume of the acid cycle.

*F*: is the Faraday constant (96,485 Cmol${}^{-1}$).

*t*: (min) is the test time.

I(A): is the current applied; where *z* is the ion’s absolute valence (*z* = 1).

*N*: is the number of repeating units (*N* = 1).

The energy consumption E(kWh/kg) was calculated by Equation (9) [33].

(9) $$E\left(\frac{kWh}{kg}\right)=\frac{\int_{0}^{t}UIdt}{(C_{t}-C_{0})VM}$$

*U*: is the voltage drop across the BMED stack (*V*).

*I*: is the current (*A*).

*t*: (min) is the test time.

*M*: is the molecular weight of salicylic acid 138,122 (g/mol).

It is important to note that organic solutions are poor electrical conductors and can cause significant drops in electrical potential in BMED cells, causing higher energy consumption in said cell.

However, a limitation is that the products to be treated by BMED must have a high mineral content to allow good electrical conductivity. This is in order to decrease the overall resistance of the electrodialysis cell [34].

### 2.7. Experimental Design Based on Taguchi Method

The Taguchi method was used to design the experiments. The Taguchi method applies fractional factorial experimental designs, called orthogonal arrays, to reduce the number of experiments. The selection of a suitable orthogonal array depends on the number of control factors and their levels. The factors and their levels are presented in Table 2.

In this work, the effect of four important factors, including electric potential, initial concentration of the salicylic acid, the concentration of the salt, and the volumetric flow of recirculation of the solutions and each factor was studied at three levels on the concentration of salicylic acid. The design of the experiment using the Taguchi method provides a simple, efficient, and systematic approach to determine the optimum conditions [35]. With the selection of the $L_{9}$ ($3^{4}$) orthogonal array, the number of experiments required can be reduced to nine, which should be conducted in order to study the main effects and interactions, whereas full factorial experimentation would require $3^{4}=81$ experiments.

For the membrane division voltage at 100 mA/cm${}^{2}$ is less than 1.2 V, in 0.5 M NaCl solution and 0.25 M Na${}_{2}$SO${}_{4}$ electrode rinse solution at 25 ${}^{\circ }$C [28]. In [7] mentioned that the maximum estimated voltage is 16 V, taking into account the possible losses. Then we chose an interval of 5 to 15 V to carry out our experiments.

The other parameters have been chosen by conditions of the experiment, the electric potential being the most influential factor in the process to be carried out.

BMED experiments were performed using aqueous sodium salicylate solutions. The set of factor levels to be tested, the experimental parameters and their levels are given in Table 3.

## 3. Results and Discussion

The effects or influence of the operating parameters, that is, electric potential, initial concentration of salicylic acid, concentration of salt and recirculation flow of solutions with the Taguchi method are shown in Table 4.

### 3.1. Taguchi Analysis

**Figure 6:**
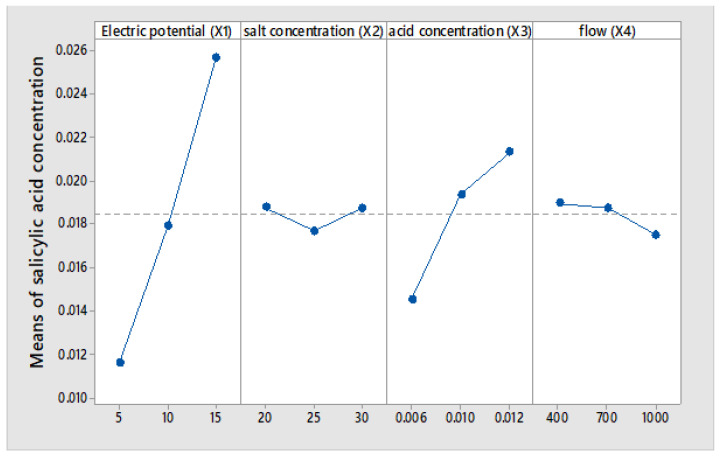
Main Effect Plot for Means of salicylic acid concentration.

**Figure 7:**
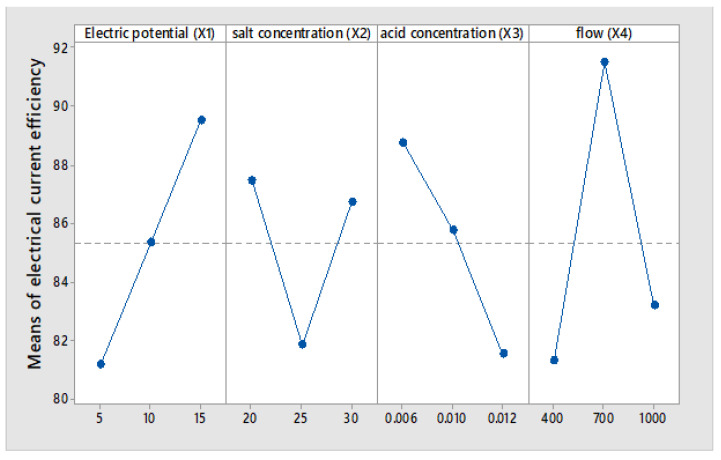
Main Effect Plot for Means of electrical current efficiency.

**Figure 8:**
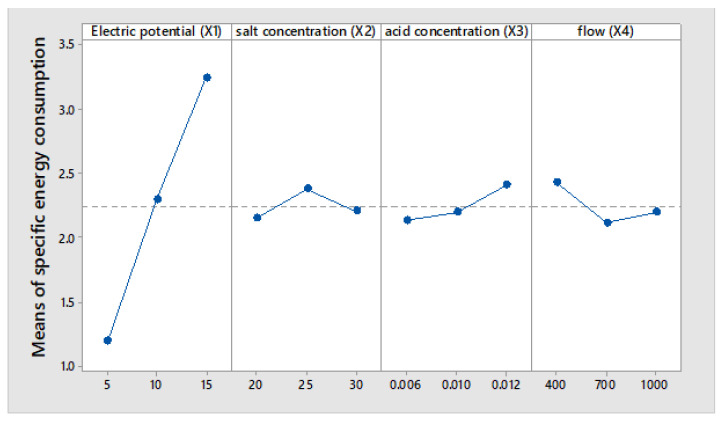
Main Effect Plot for Means of specific energy consumption.

The Taguchi method produces the mean of means results, as indicated in Figure 6, Figure 7 and Figure 8, respectively.

Main effects plots are presented for data averages of: salicylic acid concentration, Electric current efficiency, specific energy consumption caused by electric potential ($X_{1}$), salt concentration ($X_{2}$), initial acid concentration ($X_{3}$) and flow ($X_{4}$).

### 3.2. Effects of Operating Conditions on the Response Parameters

#### 3.2.1. Effect of Applied Voltage ($X_{1}$)

Three electrical potentials: 5, 10 and 15 V, were applied to the BMED cell to investigate the effect of voltage in the processing of salicylic acid. The results presented in Figure 6 show that an increase in applied voltage leads to a higher rate of acid production; the concentration of salicylic acid has a linear relationship of increase with the potential applied to the electrodialysis cell, and the average at 5 volts reaches a concentration of 0.0536 N and at 15 volts of 0.0256 N.

Voltage is the driving force of BMED. Increasing the voltage obviously improves ion transport across membranes and increases the rate of dissociation of water in the bipolar membrane [36]. However, energy consumption must be taken into account on a commercial scale. It should be noted that increasing the voltage to a limit value can burn the membranes and lead to the electrolysis of the solution, as well as high energy consumption [26].

The results presented in Figure 7 indicate that the average electric current efficiency is 85.3%, and it is observed that with the increase in the electric potential, the efficiency increases linearly at 15 volts, and 90% electric efficiency is reached. The results presented in Figure 8 show that the energy consumed to produce one kilogram of salicylic acid increases linearly with the voltage applied to the electrodialysis cell. The average at 5 volts achieves an energy consumption of 1.197 kWh/kg of salicylic acid compared to 3.241 kWh/kg. The reason is that, as the voltage drop increases, a greater part of the energy is consumed to overcome the electrical resistance [25].

#### 3.2.2. Effect of Sodium Salicylate Concentration on the Production of Salicylic Acid ($X_{2}$)

The effect of the concentration of the sodium salicylate salt at concentrations of 20, 25 and 30 g/L on the production of salicylic acid was investigated. In Figure 6 it is observed that the concentration of the acid produced does not have significant changes with the increase in the concentration of sodium salicylate.

From Figure 7, it is observed that the electric current efficiency increases in the concentration range of 25 to 30 g/L of the salt, and decreases in the range 20 to 25 g/L. The increase in current efficiency can be explained by the increase in salt concentration, which prevents the electrolysis of water on the surface of the membrane [37]. When the feed concentration exceeds 20 g/L, it is possible a part of the electrical energy is used to produce heat instead of ion migration, so that the current efficiency decreases.

From Figure 8 it is observed that when the salt concentration is at the low and high levels, the energy consumption is below the average that indicates 2.2 kWh/kg of salicylic acid. Regardless of other working conditions in [38] carried out in experiments with sodium salicylate solutions (SANa) by the cation exchange membrane process, they produced salicylic acid with a current efficiency close to 90% and an energy consumption of around 10 kWh/kg SAH produced.

#### 3.2.3. Effect of the Initial Concentration of Salicylic Acid ($X_{3}$)

The effect of the initial concentration of aqueous solutions of salicylic acid with concentrations of 0.006 M, 0.01 M and 0.012 M on the production of salicylic acid was investigated. In Figure 6 it is observed that the concentration of the acid produced increases significantly in the concentration range of 0.01–0.012 mol/L; this explains that, for very low concentrations, the aqueous solution of salicylic acid has low electrical conductivity.

The results presented in Figure 7 show that the efficiency of the electric current decreases as the initial concentrations of salicylic acid increase. At a concentration of 0.006 M, an average electric current efficiency of 89% is observed. At a concentration of 0.012 N, an average electric current efficiency of 81.52% is obtained. The energy consumption seems to be independent of the initial concentration of salicylic acid. However the results presented in Figure 8 show that the energy consumption at a concentration of 0.006 N is 2.1 kWh/kg and at a concentration of 0.012 N is 2.5 kWh/kg of acid, respectively.

#### 3.2.4. Effect of Flow ($X_{4}$)

The flow rates with which the electrolytes were circulated on the BMED module varied between 400, 700 and 1000 mL/min. The results presented in Figure 6 show that it does not present very significant changes in the three levels of flow experimentation; however, it is observed that the lower the flow, the higher the concentration of salicylic acid is reached. Increasing the flow rate decreases the residence time in the electrodialysis cell, and increasing the residence time is equivalent to improving the effective contact area of the membrane with the solutions.

Figure 7 shows that increasing the flow rate from 400 mL/min to 700 mL/min in the BMED cell results in an increase in the efficiency of the electric current by 10%. Increasing the flow rate may increase the linear velocity, which reduces the electrical resistance of the BMED cell. When the flow rate is increased from 700 to 1000 mL/min, the current efficiency decreases by 8% due to the reduction of the residence time.

From Figure 8 it is observed that the energy consumption does not present significant changes with the change of flow, however, a lower energy consumption of 2 kWh/kg of salicylic acid is observed at 700 mL/min.

### 3.3. Anova Results

The purpose of analysis of variance (ANOVA) is to investigate which process parameter significantly affected the response. The ANOVA evaluation also allows a greater understanding of whether the obtained findings are acceptable and whether the experiments are carried out under controlled conditions [39].

The analysis of variance of the salicylic acid concentration, electric current efficiency, specific energy consumption and the percentage contribution of each parameter is shown in Table 5.

According to these results, it is found that the electric potential applied to the BMED cell is the most significant factor affecting the concentration of salicylic acid, with a contribution of 78.93%. It is observed that the most significant factor that contributes to the efficiency of the electric current is the volumetric flow at 42.41% and to a lesser degree the electric potential with 25.05%. The factor that contributes to lower energy consumption is the electrical potential, which reached 94.64%.

The results presented in Table 6 show the delta values, which is the difference between the maximum and minimum values obtained for each factor, allowing the factors to be organized in order of significant influence regarding the response.

Regarding the concentration of the salicylic acid produced, the following are obtained: $X_{1}$, $X_{3}$, $X_{4}$ and $X_{2}$ in descending order; electric current efficiency $X_{4}$, $X_{1}$, $X_{3}$ and $X_{2}$; electric power consumption $X_{1}$, $X_{4}$, $X_{3}$ and $X_{2}$.

**Figure 9:**
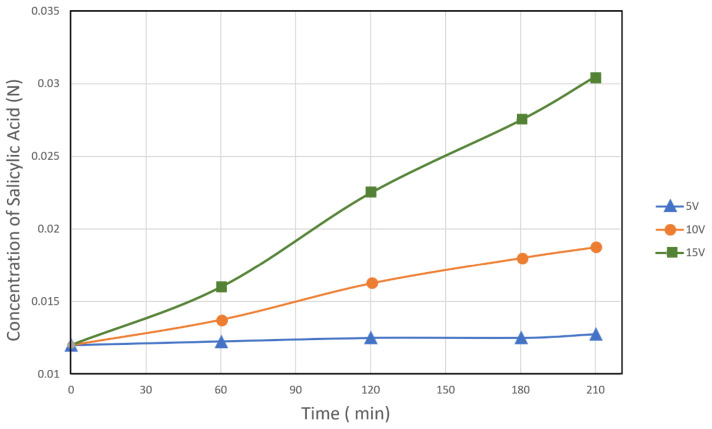
Salicylic acid concentration in the acid compartment at various electric potentials as a function of time.

**Figure 10:**
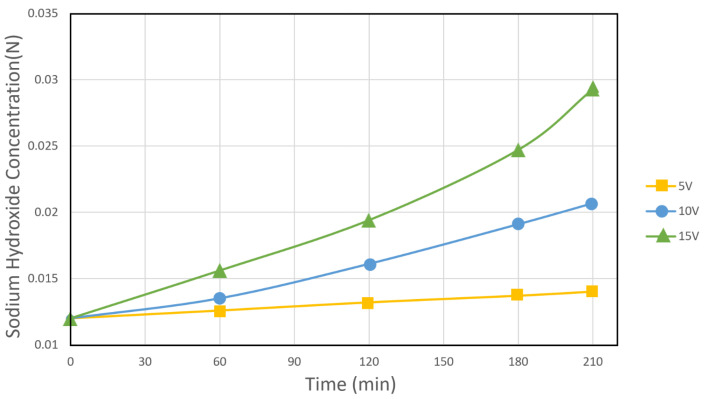
Sodium hydroxide concentration at various electric potential as a function of time.

The results presented in Figure 9 and Figure 10 show that the concentration of salicylic acid and sodium hydroxide varies linearly with the operating time of the electrodialysis cell, regardless of the applied voltage. An increase in the potential to the cell from 5 to 15 V leads to a higher concentration of acid and base; at approximately 0.30 mol/L of salicylic acid and 0.29 mol/L of sodium hydroxide occurs at a potential of 15 Volts.

These values are very similar to those reported in [5] working at different current densities such as: 30, 50 and 75 mA/cm${}^{2}$ with an initial concentration of salicylic acid of 2 g/L. The following values were obtained as salicylic acid concentration: 3.4, 3.0 and 4.6 g/L in an operating time of 30 min as indicated in Table 7.

In [40], it was mentioned that the energy consumption of the BMED process for the production of gluconic acid increases almost linearly with the increase in current. The increasing trend of power consumption is consistent with Ohm’s law. Being works with productions of different substances, there is a certain similarity in the experimental data obtained in [40] as shown in Table 8.

As for the electric current efficiency values (η), they are very similar to those reported in [38]. As shown in Table 9.

The research work developed is not within the scope of evaluating the production cost of salicylic acid. However, in [40] it was mentioned that the cost of the energy consumption increases linearly with the increase in current. The contribution to the total cost of the process increases from 13 to 73% when the current increases from 10 to 50 A. Then in [41] indicated that the total investment is the sum of the cost of the battery and the cost of the peripheral equipment.

## 4. Conclusions

A laboratory scale experimental setup was used to verify the viability of BMED to produce salicylic acid from sodium salicylate. An electrodialysis module has been constructed with commercial cationic, anionic and bipolar exchange membranes for the conversion of sodium salicylate to salicylic acid.

The effect of operating conditions, such as applied electrical potential, salt concentration, initial acid concentration, and volumetric flow on BMED yields was investigated. The performance of the module was quantified in terms of salicylic acid concentration, electric current efficiency and specific energy consumption (kWh/kg of salicylic acid), as shown in the figures in Section 3.1.

Taguchi’s methodology shows us that in Figure 6, the electric potential of the cell and the initial acid concentration are the most representative parameters of the salicylic acid production process. The results observed in Figure 7 and Figure 8 indicate average electric current efficiencies of 85.3%, and the specific energy consumption is 2.24 kWh/kg of salicylic acid, respectively.

The experimental results indicate that the four-compartment BMED process can be applied to prepare salicylic acid and sodium hydroxide, and will also achieve environmental benefits.

## Figures and Tables

**Table 1:** Membrane characteristics.

Types of Membranes	CEM	AEM	FBM [27,28]
Electric resistance (Ωcm${}^{2}$).	2.50–3.50	2.50–3.50	<3
Exchange capacity (meq/g)	1.50–1.80	1.40–1.70	
Thickness (mm)	0.17–0.19	0.16–0.18	0.13–0.16

**Table 2:** Parameters and their values corresponding to their levels studie in experiments.

N${}^{\circ }$	Factors	Notation	Unit	Levels		
				Low	Medium	High
1	Voltage supplied	$X_{1}$	V	5	10	15
2	Salt concentration	$X_{2}$	g/L	20	25	30
3	Initial concentration of salicylic acid	$X_{3}$	M	0.006	0.010	0.012
4	Volumetric flow	$X_{4}$	mL/min	400	700	1000

**Table 3:** Taguchi $L_{9}\left(3^{4}\right)$.

Run	Operating Parameters			
	$X_{1}$	$X_{2}$	$X_{3}$	$X_{4}$
1	5	20	0.006	400
2	5	25	0.010	700
3	5	30	0.012	1000
4	10	20	0.010	1000
5	10	25	0.012	400
6	10	30	0.006	700
7	15	20	0.012	700
8	15	25	0.006	1000
9	15	30	0.010	400

**Table 4:** Response table.

Run	Operating Parameters				Responses			
	$X_{1}$	$X_{2}$	$X_{3}$	$X_{4}$	Salicylic Acid Concentration (M)	Salicylic Acid Concentration (g/L)	Electrical Efficiency % (η)	Energy Consumption (E kWh/kg)
1	5	20	0.006	400	0.0087	1.2016	82.70	1.173
2	5	25	0.010	700	0.0122	1.6850	84.24	1.151
3	5	30	0.012	1000	0.0140	1.9330	76.58	1.267
4	10	20	0.010	1000	0.0184	2.5410	85.73	2.111
5	10	25	0.012	400	0.0207	2.8590	74.03	2.767
6	10	30	0.006	700	0.0148	2.0440	96.28	2.015
7	15	20	0.012	700	0.0293	4.0460	93.96	3.180
8	15	25	0.006	1000	0.0202	2.7990	87.27	3.212
9	15	30	0.010	400	0.0275	3.7980	87.25	3.330

**Table 5:** ANOVA for acid concentration, electrical efficiency and energy consumption.

Variables Responses	Factor	DOF	Sum of Squares	Contribution(%)	SS Ajust	Mc ajus
Concentration of Salicylic acid	$X_{1}$	2	29.6 $\times \;10^{-5}$	78.93	0.3 $\times \;10^{-3}$	14.8 $\times \;10^{-5}$
	$X_{2}$	2	0.2 $\times \;10^{-5}$	0.5	0.3 $\times \;10^{-3}$	0.1 $\times \;10^{-5}$
	$X_{3}$	2	7.3 $\times \;10^{-5}$	19.43	0.7 $\times \;10^{-4}$	3.6 $\times \;10^{-5}$
	$X_{4}$	2	0.4 $\times \;10^{-5}$	1.06	0.4 $\times \;10^{-5}$	0.2 $\times \;10^{-5}$
	Total	8	37.5 $\times \;10^{-5}$	100		
Electric current efficiency	$X_{1}$	2	103.834	25.05	103.834	51.9170
	$X_{2}$	2	55.712	13.44	55.712	27.8559
	$X_{3}$	2	79.065	19.08	79.065	39.5325
	$X_{4}$	2	175.736	42.41	175.736	87.8678
	Total	8	414.346	100		
Specific energy consumption	$X_{1}$	2	6.27729	94.64	6.27729	3.13864
	$X_{2}$	2	0.08153	1.220	0.08153	0.04077
	$X_{3}$	2	0.12070	1.810	0.12070	0.06035
	$X_{4}$	2	0.15286	2.300	0.15286	0.07643
	Total	8	6.63238	100		

**Table 6:** Order of influence of response parameters.

Variables Responses	Levels	Factors			
		$X_{1}$	$X_{2}$	$X_{3}$	$X_{4}$
Concentration of salicylic acid	1	0.01163	0.01880	0.01457	0.01897
	2	0.01797	0.01770	0.01937	0.01877
	3	0.02567	0.01877	0.02133	0.01753
	Delta	0.01403	0.00110	0.00677	0.00143
	Sort out	1	4	2	3
Electric current efficiency	1	81.17	87.46	88.75	81.33
	2	85.35	81.85	85.74	91.49
	3	89.49	86.70	81.52	83.19
	Delta	8.32	5.62	7.23	10.17
	Sort out	2	4	3	1
Specific energy consumption	1	1.197	2.155	2.133	2.423
	2	2.298	2.377	2.197	2.115
	3	3.241	2.204	2.405	2.197
	Delta	2.044	0.222	0.271	0.308
	Sort out	1	4	3	2

**Table 7:** Comparative data for salicylic acid concentration and current density.

Experiment	Electric Potential (V)	Salicylic Acid Concentration (g/L)	Density of Electric Current (mA/cm${}^{2}$) [5]	Salicylic Acid Concentration (g/L) [5]
1	5	1.606	30	3.4
2	10	2.481	50	4.0
3	15	3.547	75	4.6

**Table 8:** Comparative data of Energy consumption (E).

Experiment	Electric Potential (V)	Energy Consumption (kWh/kg)	Electric Current Intensity (A) [40]	Energy Consumption (kWh/kg) [40]
1	5	1.197	10	1.2
2	10	2.297	20	2.1
3	15	3.240	30	3.0

**Table 9:** Comparative data of Electrical efficiency (η).

Experiment	Electric Potential (V)	η	Density of Electrical Current (mA/cm${}^{2}$) [38]	η [38]
1	5	81.17	2.0	86.80
2	10	85.34	5.0	89.41
3	15	88.82	7.5	51.84

## Data Availability

Not applicable.

## Abbreviations

The following abbreviations are used in this manuscript:

MDPI	Multidisciplinary Digital Publishing Institute
DOAJ	Directory of open access journals
TLA	Three letter acronym
LD	Linear dichroism
